# Network Pharmacology-Based Analysis of the Underlying Mechanism of *Hyssopus cuspidatus* Boriss. for Antiasthma: A Characteristic Medicinal Material in Xinjiang

**DOI:** 10.1155/2021/7671247

**Published:** 2021-11-29

**Authors:** Rongchang Liu, Yan Mao, Zhengyi Gu, Jinhua He

**Affiliations:** ^1^College of Pharmacy, Xinjiang Medical University, Urumqi 830011, China; ^2^Xinjiang Institute of Materia Medica, Urumqi 830004, China

## Abstract

**Background:**

*Hyssopus cuspidatus* Boriss. (Shen Xiang Cao (SXC)), a traditional medicine herb in Xinjiang, has a long history of being used by minorities to treat asthma. However, its active antiasthmatic compounds and underlying mechanism of action are still unknown. The aim of this study was to investigate the bioactive compounds and explore the molecular mechanism of SCX in the treatment of asthma using network pharmacology.

**Methods:**

The compounds of SCX were collected by a literature search, and Traditional Chinese Medicine Systems Pharmacology Database and Analysis Platform (TCMSP) and SwissTargetPrediction were used to predict targets and screen active compounds. Moreover, asthma-related targets were obtained based on DisGeNET, Herb, and GeneCards databases, and a protein-protein interaction (PPI) network was built by the STRING database. Furthermore, the topological analysis of the PPI and SXC-compound-target networks were analyzed and established by Cytoscape software. Finally, the RStudio software package was used for carrying out Gene Ontology (GO) function enrichment and Kyoto Encyclopedia of Genes and Genomes (KEGG) pathway analysis. AutoDock tools and AutoDock Vina were used to molecularly dock the active compounds and key targets.

**Results:**

A total of 8 active compounds and 258 potential targets related to SXC were predicted, and PPI network screened out key targets, including IL-6, JUN, TNF, IL10, and CXCL8. GO enrichment analysis involved cell responses to reactive oxygen species, oxidative stress, chemical stress, etc. In addition, KEGG pathway analysis showed that SXC effectively treated asthma through regulation of mitogen-activated protein kinases (MAPK) signaling pathways, interleukin 17 (IL-17) signaling pathways, toll-like receptor (TLR) signaling pathways, and tumor necrosis factor (TNF) signaling pathways.

**Conclusion:**

The preliminary study that was based on multiple compounds, multiple targets, and multiple pathways provides a scientific basis for further elucidating the molecules involved and the underlying antiasthma-related mechanisms of SXC.

## 1. Introduction

Asthma is a type of respiratory tract allergic inflammatory disease in which mast cells and airway epithelial cell dysfunction play an important role [1, 2]. The predominant clinical symptoms of asthma include chest tightness, coughing, wheezing, and shortness of breath [3]. Although corticosteroids are considered as the first-line clinical drug to relieve the symptoms of patients [4], it cannot completely cure, or even aggravate symptoms of asthma sometimes [5]. In recent studies, it has been shown that asthma is likely related to epigenomes and environmental factors [6, 7]. According to a national cross-sectional study, it was estimated that there are currently about 47.5 million patients with asthma in China and that some patients have not yet been effectively diagnosed and treated [8]. Furthermore, the incidence of asthma in Chinese children aged 0–14 years is gradually increasing [9]. The complex pathogenesis of asthma is still unclear. At present, it is widely believed that the balances of Th1/Th2 and TH17/Treg play an important role in the pathogenesis of asthma. [10]. Th2 cells can release various cytokines, such as IL-4, IL-5, and IL-13. These cytokines could drive the recruitment of eosinophil cells to accumulate in the lung [11]. The cytokine, IFN-*γ*, produced by Th1 cells can inhibit Th2 cell proliferation, induce eosinophil apoptosis, and antagonize the inflammatory factor [12]. Th17 produces various cytokines including IL-17, IL-6, and TNF-*α* [13]. TNF-*α* is a key inflammatory mediator that is produced by macrophages. It is a regulatory factor in the immune response and in cell proliferation and differentiation [14]. IL-6 is a proinflammatory cytokine produced after immune activation that can aggravate chronic inflammation [15].

Traditional medicine in Xinjiang is an important medicinal system of Traditional Chinese Medicine (TCM), and it is one of the part of medicines in China. Shen Xiang Cao (SXC, Labiatae family) comes from the dried ground aerial parts of *Hyssopus cuspidatus* Boriss. and is mainly distributed in Altay, Tianshan, and Kunlun Mountains [16, 17].

Previous studies performed by Jinhua He's team showed that different elution (water and ethanol) of SXC has a certain antagonistic effect on the acetylcholine-induced or histamine-induced smooth muscle contraction of isolated tracheal in guinea pigs, and 60% ethanol elution shows the strongest effect, which has similar effects with aminophylline at high mass concentration [18]. Furthermore, an ethanol extract of SXC has an anti-inflammatory effect and inhibits the production of inflammatory-related factors in RAW264.7 cells and activation of the NF-*κ*B signaling pathway and MAPK signaling pathway [19]. Moreover, it was found that SXC has a certain regulatory effect on Th1 and Th2 immune responses as well as a relaxing effect on bronchial smooth muscle, which may have an antagonistic effect on the M cholinergic receptor and H1 receptors [20]. As a traditional folk medicine, SXC is frequently used by Xinjiang minorities to treat cough, asthma, and wheezing [21]. However, the basis of the compound and the underlying antiasthma mechanism of action are still unclear. Therefore, the aim of this study was to research the targets and mechanisms of action of the active ingredients of SXC with the overall perspective of network pharmacology to explore the underlying mechanism of SXC in the antiasthma process.

## 2. Materials and Methods

### 2.1. Collection of Bioactive Compounds and Targets

“Hyssopus cuspidatus Boriss.” was used as a key search term in the China National Knowledge Infrastructure (CNKI) database (https://www.cnki.net/), Wanfang database (http://www.wanfangdata.com.cn/index.html), Weipu database (http://www.cqvip.com/), and PubMed database (https://pubmed.ncbi.nlm.nih.gov/) to obtain chemical compounds of SXC. Subsequently, TCMSP (https://tcmspw.com/tcmsp.php) were adopted to screen compounds with conditions of oral bioavailability (OB) ≥ 30% [22] and drug-like (DL) activity ≥0.18 [23] and collect compounds-related targets.

In order to obtain more targets, simplified molecular input line entry specification (SMILES) information of bioactive compounds was obtained from the PubChem (https://pubchem.ncbi.nlm.nih.gov/) database and we imported SMILES information into the SwissTargetPrediction platform (http://www.swisstargetprediction.ch/), and the attribution was set as “homo sapiens” and probability ≥0.4 to collect the target of the compound.

### 2.2. Collection of Targets of Asthma

The word “asthma” was used as a keyword in the GeneCards database (https://www.genecards.org/), Herb database (http://herb.ac.cn/), and DisGeNET database (https://www.disgenet.org) and the attribute was set to “homo sapiens” to obtain targets in different databases. Subsequently, all targets were uploaded to the online Venn diagram (https://bioinfogp.cnb.csic.es/tools/venny/index.html) to obtain the common targets.

### 2.3. Establishment of an SXC-Compounds-Target Protein Network

The Uniprot database (https://www.uniprot.org/) was applied to normalize the names of target genes and convert it into abbreviations, and targets without corresponding gene names were deleted. Finally, the obtained chemical compounds and their corresponding targets were imported into Cytoscape (version 3.8.0 for Windows) software to construct a network visual diagram of SXC-compounds-targets.

### 2.4. Establishment of Protein Interaction Network

The targets of compounds and disease were uploaded to the online Venn diagram, respectively, to obtain their intersection genes, which data were sequentially uploaded to the STRING 11.0 database (https://string-db.org/). Species was set to “homo sapiens” and confidence level was set to ≥0.700, respectively. After deleting the free target, a target-protein interaction network was constructed. Finally, the data were imported into Cytoscape software for visual analysis and network topology analysis, and key target analysis was performed through plug-in CytoNCA to obtain the PPI network.

### 2.5. Analysis of GO and KEGG Enrichment

GO biological function enrichment analysis and KEGG signaling pathway enrichment analysis on the key genes of PPI by using R (version 4.0.3 for Windows) and RStudio (version 1.4.1103 for Windows) software which install the packages of org.Hs.eg.db, ggplot2, enrichplot, and clusterProfiler [24]. To understand the complex relationships among compounds, targets, and pathways, compound-target-pathway networks were constructed and analyzed by Cytoscape.

### 2.6. Molecular Docking

In preparation for molecular docking, the three-dimensional (3D) protein structures of the key proteins in the PPI network and four active chemical ingredients were downloaded from the Protein Data Bank (PDB) database (http://www.rcsb.org/) and PubChem database, respectively. Then, budesonide was considered as a reference [25], and the SDF format of the compound was converted into the MOL2 format using Open Babel software (http://openbabel.org/wiki/Main_Page). After dehydration, hydrogenation, and compound pretreatment of the downloaded proteins with AutoDock tools, AutoDock Vina (version 1.1.2 for Windows) software (http://vina.scripps. edu/) was used for molecular docking of the receptor and ligand. Finally, visualizing and analyzing the results of molecular docking conformation were completed by PyMOL (version 2.4.1 for Windows) software (https://pymol.org/2/) and ProteinsPlus (https://proteins.plus/).

## 3. Results and Discussion

### 3.1. Active Compounds and Targets of SXC

By a literature search, the composition database of SXC was established; all compounds were screened by the TCMSP database for OB ≥ 30% and DL ≥ 0.18. To further discover the targets, the corresponding targets of these bioactive compounds information were supplemented with SwissTargetPrediction database. After deleting the duplicate targets, a total of 257 potential targets and 8 bioactive compounds were found (Table 1).

### 3.2. Asthma-Related Targets and Common Targets

**Figure 1:**
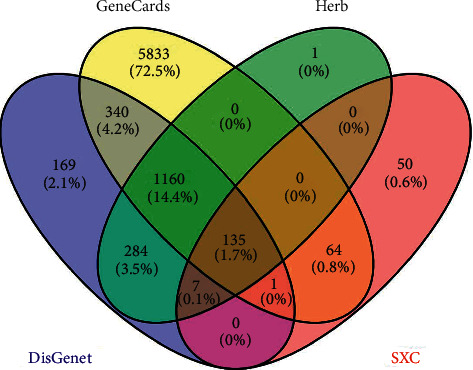
Venn diagram of targets of SXC and asthma.

The DisGeNET database screened 2096 targets, the GeneCards database screened 7533 targets, and the Herb database screened 1587 targets. Gene names of the above targets were included in the online Venn diagram and a total of 1295 asthma-related intersection targets was obtained. Then, asthma-related targets and targets of active compounds were uploaded to the online Venn diagram and 135 common genes were obtained between SXC and asthma (Figure 1).

### 3.3. Construction and Analysis of SXC-Compounds-Target Network

**Figure 2:**
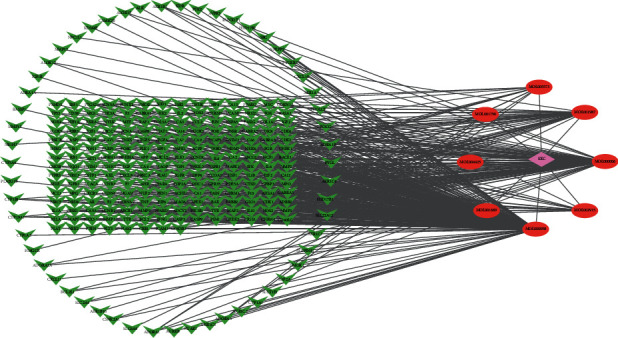
SXC-compounds-target network (red ellipses represent compounds, and green V represents compounds, and pink diamond represents SXC).

A total of 8 active chemical compounds of SXC and their corresponding targets were imported into Cytoscape software, and the SXC-compound-target network relationship diagram (Figure 2) was obtained. Figure 2 shows that the diagram included a total of 268 nodes and 407 edges. The results indicated that quercetin (degree = 214.0), luteolin (degree = 92.0), *β*-sitosterol (degree = 37.0), acacetin (degree = 29.0), salvigenin (degree = 15.0), and genkwanin (degree = 13.0) may be had relationship in the treatment of asthma.

### 3.4. PPI Network Construction and Topology Analysis

**Figure 3:**
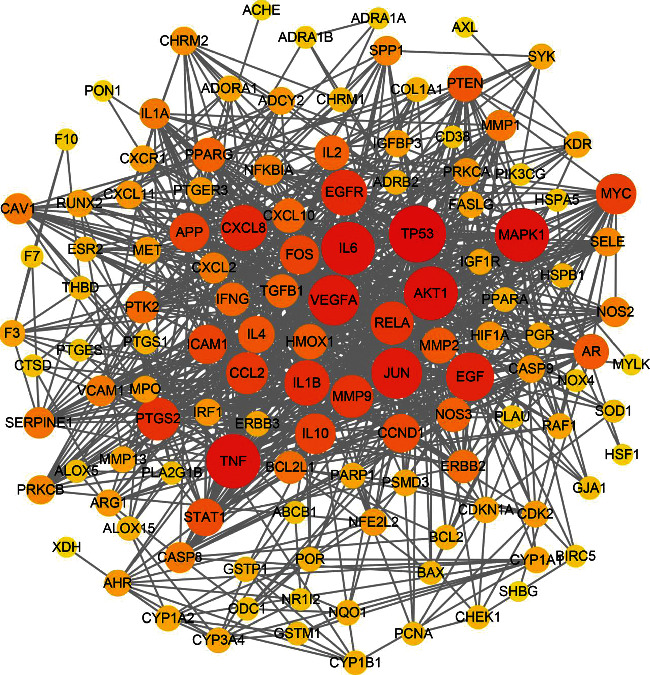
The protein-protein interaction (PPI) network (the size and color saturation of the node were proportional to its significance).

**Figure 4:**
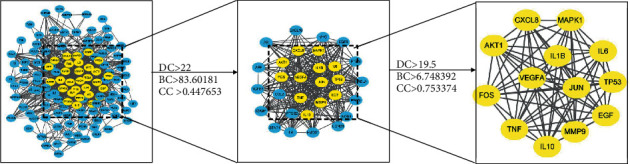
Key targets screening process.

After importing the common targets to the STRING database, the subnetworks that cannot be connected to the free target and the main network were deleted. The PPI network was imported into Cytoscape software for key target analysis, and 125 nodes and 983 edges were obtained (Figure 3). Then, CytoNCA plug-in was performed to obtain all targets. The top 5 targets in degree value were IL-6 (degree = 12.0), EGF (degree = 12.0), JUN (degree = 12.0), TNF (degree = 12.0), and IL-10 (degree = 11.0). The average degree of node is 3.03 and average of closeness is 0.40, and the average of betweenness is 405.97. Colors and size indicated that the importance of target in the network. If the degree (DC) and betweenness (BC) were greater than twice the median value and if closeness (CC) was greater than the median value to screen key targets [30], construction and topology analysis of the PPI network was performed as shown in Figure 4 and Table 2.

### 3.5. Analysis of GO Function Enrichment and KEGG Pathway Enrichment

**Figure 5:**
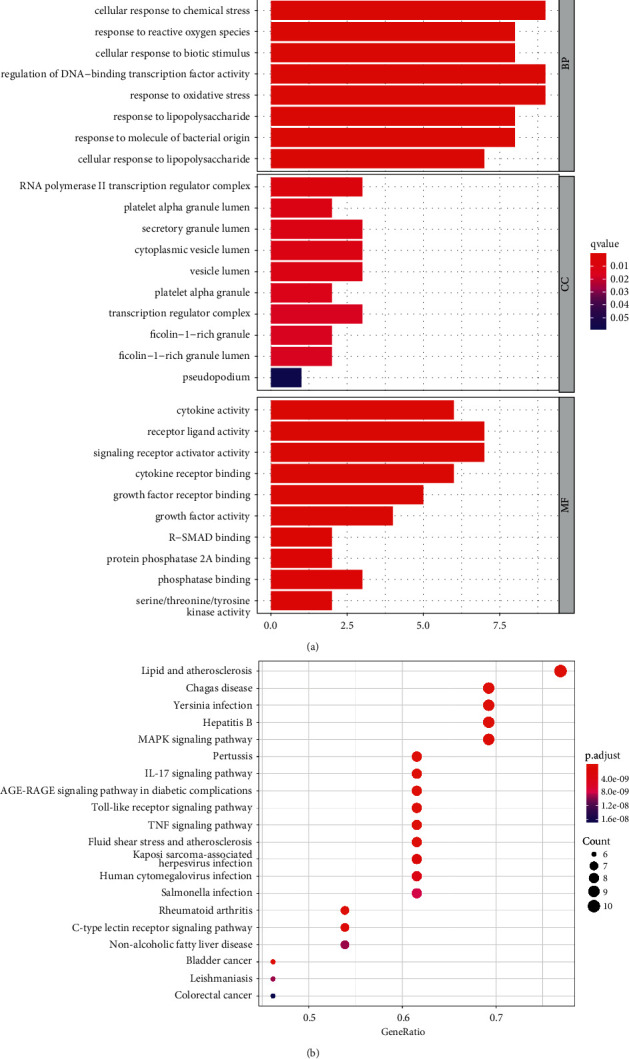
GO and KEGG pathway enrichment analyses.

The key targets were analyzed by RStudio software, and a total of 1677 GO entries with *P* < 0.05 were enriched (Figure 5). This involved 1606 biological process (BP) that included cellular responses to reactive oxygen species (ROS) and cellular responses to oxidative stress and chemical stress. Furthermore, 9 cellular components (CC) were found to contain a platelet alpha granule lumen and a secretory granule lumen. In addition, 62 molecular functions (MF) were found that mainly include receptor ligand activity, signal receptor activator activity, and cytokine receptor binding.

A total of 110 pathways with *P* < 0.05 were enriched by KEGG pathway enrichment, and Figure 5 showed the bubble chart of the top 20 KEGG signaling pathway enrichment analysis. The asthma-related pathways mainly included advanced glycation end products (AGE)-receptor for advanced glycation end products (RAGE) signaling pathway, IL-17 signaling pathway, TNF signaling pathway, and MAPK signaling pathway. In addition, other diseases were involved, such as Chagas disease, whooping cough, Yersinia infection, and hepatitis B.

### 3.6. Compounds-Target-Pathway Network

**Figure 6:**
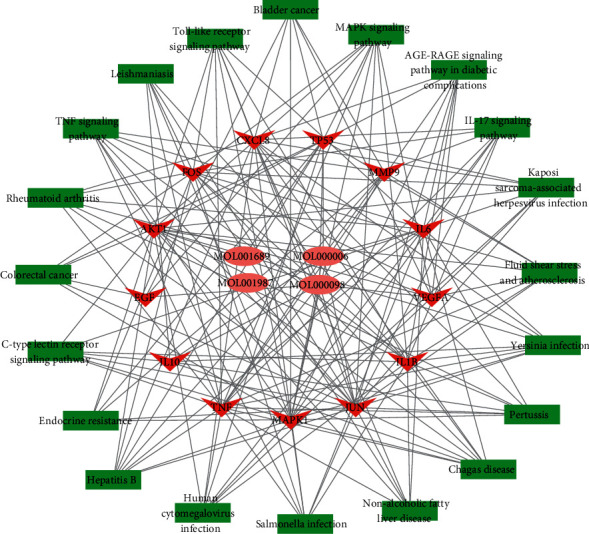
Network of active compounds-targets-pathway (pink ellipses represent compounds, red V-grooves represent target, and green rectangles represent pathways).

As shown in Figure 6, Cytoscape was used to construct a compounds-targets-pathway network. The network consisted of 38 nodes and 183 edges, including 4 active compounds, 13 targets, and 20 KEGG pathways. These findings indicate that SXC acts on multiple pathways through multiple targets of multiple compounds to achieve a therapeutic effect on asthma.

### 3.7. Diagram of the Treatment Pathway of SXC

**Figure 7:**
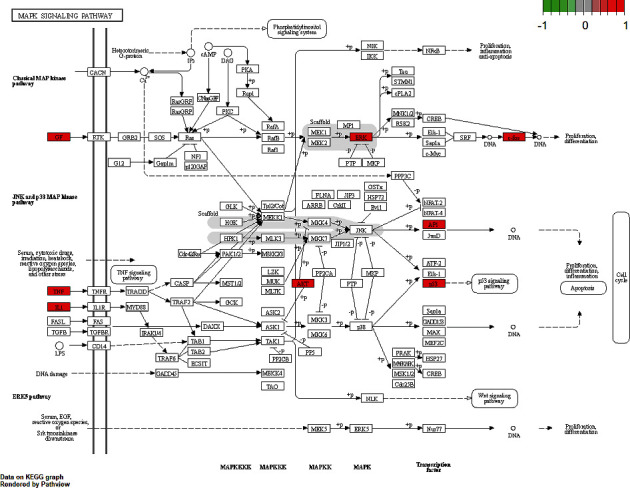
MAPK signaling pathway enrichment analysis (red rectangles represent important targets).

KEGG Mapper was applied to display the key targets in the pathways that were most related to asthma. As shown in Figure 7, red squares represent pathway network multiple nodes that are targets of the compounds that can be regulated by quercetin, luteolin, and *β*-sitosterol. All abovementioned results demonstrated that the active ingredients of SXC achieved effects through multiple targets and pathways and synergistic effects.

### 3.8. Molecular Docking

**Figure 8:**
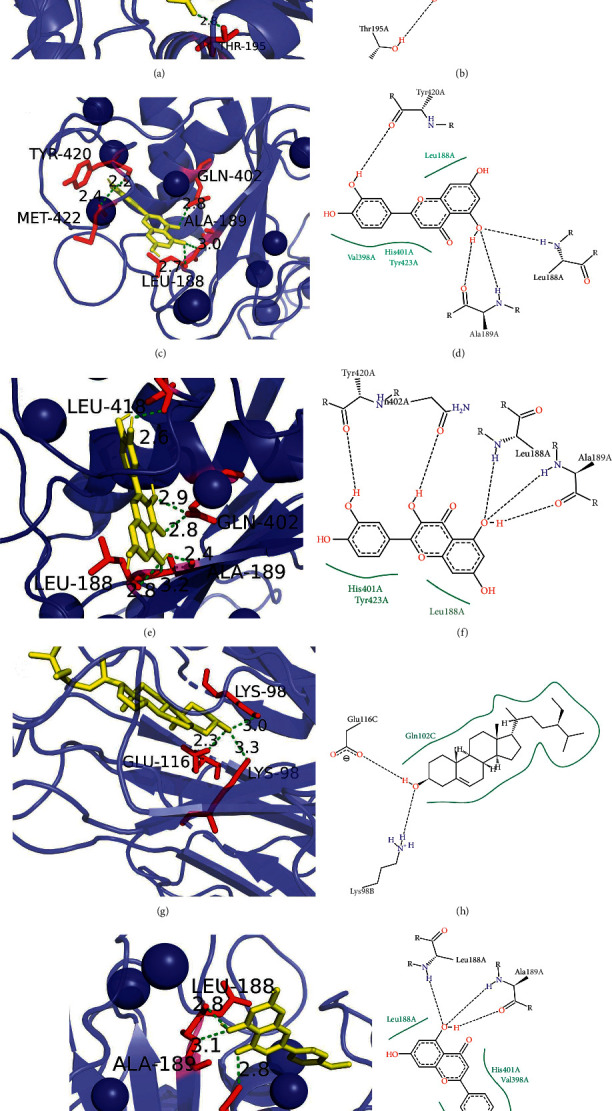
Docking binding energy between ligand and protein. (a, b) Budesonide-AKT1. (c, d) Luteolin-MMP9. (e, f) Quercetin-MMP9. (g, h) Beta-sitosterol-TNF. (I, j) Acacetin-MMP9.

The top 4 active ingredients with a degree value of SXC were selected as ligands, while 13 core targets of the PPI network were used as receptors. To further validate the SXC compounds against asthma, molecular docking was carried out using the abovementioned ligands and receptors. The results of molecular docking showed that luteolin, quercetin, acacetin, and *β*-sitosterol all bound well to TNF, MMP9, and AKT1 (Figure 8 and Table 3).

## 4. Discussion

SXC from Xinjiang, China, has a long-term effect and shown significant treatment efficacy and regional and national features to treating certain diseases. For example, Loki Zupa Decoction [31] and Hanchuan Zupa Granule [32] both are related to SXC for the treatment of asthma. Network pharmacology results showed that 4 active ingredients of SXC, most of them belonging to flavonoids, may be related to the underlying antiasthmatic mechanism of action. Furthermore, in previous studies, it was shown that flavonoids have antitumor, antioxidation, anti-inflammatory, and anticoagulation effects and improve sugar and lipid metabolism [33]. The results of the compounds-target-pathway diagram demonstrated that quercetin, luteolin, *β*-sitosterol, and acacetin highly correlated with antiasthma effects.

As a natural flavonoid with polyphenolic hydroxyl groups, luteolin can alleviate the inflammatory response in a variety of ways [34] and reduce the production of reactive oxygen and reactive nitrogen, thereby affecting the metabolism of arachidonic acid and various inflammatory signaling pathways [35] and inhibiting the expression of inflammatory cytokines and inflammatory mediators [36]. Quercetin is a flavonol compound that is widely distributed in plants, vegetables, and fruits [37] and has a variety of biological activities against cardiovascular diseases, cancer, and diabetes [38]. Several studies have shown that the effect of anti-inflammatory and antiasthma by quercetin has been verified [39–41]. In addition, in a previous report, it was demonstrated that quercetin can improve beta-agonist-induced relaxation and relax airway smooth muscle [42], which is similar to our previous study results. Moreover, in recent reports, it was shown that acacetin has anti-inflammatory [43, 44] and antiasthma [45] activity in lipopolysaccharide (LPS), D-galactosamine, and ovalbumin-induced damage. Its mechanism of action may involve inhibition of the expression of iNOS and COX-2 and phosphorylation of I*κ*B*α* [46], weakening TNF-*α*, IL-6, and TLR 4 levels and inhibiting the phosphorylation of kinases and expression of NF‐*κ*B [47].

As shown by the analysis results of the PPI network topology, 13 key targets were screened, and most were inflammatory cytokines, it is worth to note that IL-6, TNF-*α*, and IL-10 play an important role in the development of asthma. IL-6 is a cytokine that is activated and secreted by lymphocytes and mononuclear macrophages and has an important impact on the development of asthma [48]. Furthermore, IL-6 receptor signaling regulates the expression of Th17-related genes by activating STAT3 to induce the development of Th17 cells [49]. IL-10 is a multicell-derived, multifunctional anti-inflammatory cytokine that is involved in the regulation of cell growth and differentiation as well as in inflammatory and immune responses [50]. In addition, IL-10 plays a key role in controlling immune responses. As recognized inflammatory and immunosuppressive factors, the TNF family plays an important role in the pathogenesis of asthma and inflammation of other tissues [51].

The GO enrichment data showed that SXC mainly plays a role in the responses of cells to ROS, oxidative stress, chemical stress, and LPS. Obviously, there is an obvious synergy between the different signaling pathways with the enrichment of the KEGG pathway, such as TLR signaling pathway, TNF signaling pathway, and MAPK signaling pathway. It is worth noting that the MAPK signaling pathway is an important pathway involved in the pathogenesis and progression of bronchial asthma [52]. Related regulatory factors of the MAPK signaling pathway include c-Jun N-terminal kinase (JNK), p38, and extracellular signal-regulated kinase (ERK) [53]. Furthermore, the MAPK signaling pathway can control the expression of inflammatory factors (IL-6 and TNF-*α*) to regulate inflammation and immune responses in asthma [54, 55]. It is reported that the ethyl acetate extract of SXC can significantly reduce the levels of ET-1, IL-2, IL-6, and TNF-*α* in the serum of asthmatic rats in a dose-dependent trend [56]. Hou et al. [57] observed the effect of SXC on cytokines in allergic asthma mice and found that after SXC administration, the levels of IL-4 and IL-17 in BALF of asthma mice were significantly reduced, and IFN-*γ* level was significantly increased.

In this study, molecular docking was carried out to investigate the binding ability between the compounds and targets. The smaller the value of binding energy, the better binding between the compound and target; the results of molecular docking showed that the binding energy of receptor and ligand were both < −5.0 kcal/mol.

## 5. Conclusions

In summary, the results of network pharmacology showed that *Hyssopus cuspidatus* Boriss. has multiple compounds, multiple targets, and multiple pathways to synergistically play a role in treating asthma. These findings point out the direction and provide scientific evidence for subsequent clinical and experimental research. However, considering the certain limitations in network pharmacology of the databases updates and different target prediction mechanisms, it is still necessary to verify the pathway and target through the experiments in the future to confirm the accuracy of the prediction results of this study.

## Figures and Tables

**Table 1:** The information of the bioactive compounds of SXC.

No.	MOL ID	Compound	OB (%)	DL	CAS	Molecular structure	Molecular formula	Molecular weight (g/mol)	Reference
1	MOL000006	Luteolin	36.16	0.25	491-70-3	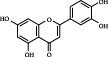	C_15_H_10_O_6_	286.24	[26]
2	MOL001689	Acacetin	34.97	0.24	480-44-4	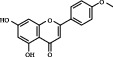	C_16_H_12_O_5_	286.26	[26]
3	MOL000098	Quercetin	46.43	0.28	117-39-5	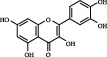	C_15_H_10_O_7_	302.23	[26]
4	MOL002915	Salvigenin	49.07	0.33	19103-54-9	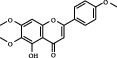	C_18_H_16_O_6_	328.30	[19]
5	MOL005573	Genkwanin	37.13	0.24	437-64-9	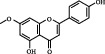	C_16_H_12_O_5_	284.26	[27]
6	MOL004425	Icariin	41.58	0.61	489-32-7	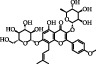	C_33_H_40_O_15_	676.70	[16]
7	MOL001987	*β*-Sitosterol	36.91	0.75	83-46-5	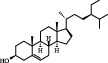	C_29_H_50_O	414.70	[28]
8	MOL001790	Linarin	39.84	0.71	480-36-4	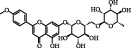	C_28_H_32_O_14_	592.50	[29]

**Table 2:** Key targets of PPI network.

No.	Target	Degree	Betweenness	Closeness
1	IL-6	12.00	1.52	1.00
2	EGF	12.00	1.52	1.00
3	JUN	12.00	1.52	1.00
4	TNF	12.00	1.52	1.00
5	IL10	11.00	0.82	0.92
6	MMP9	11.00	1.09	0.92
7	CXCL8	11.00	0.82	0.92
8	MAPK1	11.00	1.09	0.92
9	AKT1	11.00	1.07	0.92
10	VEGFA	10.00	0.89	0.86
11	FOS	10.00	0.89	0.86
12	IL1B	10.00	0.62	0.86
13	TP53	9.00	0.62	0.80

**Table 3:** Binding affinity of the compounds and key targets by molecular docking.

No.	Compound	Molecular structure	Target	PDB ID	Affinity (kcal/mol)	Polar contact	Center (*X*, *Y*, *Z*)
1	Budesonide	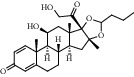	AKT1	3CQW	−9.5	ARG-4	2.68
						LYS-179	−0.87
						Thr-195	26.01
2	Luteolin	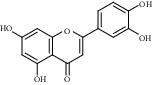	MMP9	2OW0	−10.5	LEU-188	
						ALA-189	46.89
						GLN-402	12.14
						TYR-420	49.07
						MET-422	
3	Quercetin	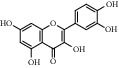	MMP9	2OW0	−10.5	LEU-188	46.89
						ALA-189	12.14
						GLN-402	49.07
4	Beta-sitosterol	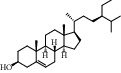	TNF	1TNF	−9.5	LEU-418	19.97
						LYS-98	49.68
						GLU-116	39.93
5	Acacetin	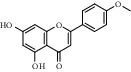	MMP9	2OW0	−10.2	ALA-189	46.89
						GLN-402	12.14
						LEU-188	49.07

## Data Availability

The data used to support the findings of this study are available from the corresponding author upon request.
